# Preliminary Outcomes of a Digital Therapeutic Intervention for Smoking Cessation in Adult Smokers: Randomized Controlled Trial

**DOI:** 10.2196/22833

**Published:** 2020-10-06

**Authors:** Jamie Webb, Sarrah Peerbux, Peter Smittenaar, Sarim Siddiqui, Yusuf Sherwani, Maroof Ahmed, Hannah MacRae, Hannah Puri, Sangita Bhalla, Azeem Majeed

**Affiliations:** 1 Digital Therapeutics Inc San Francisco, CA United States; 2 Imperial College London London United Kingdom

**Keywords:** digital, smoking, cessation, mobile, randomized controlled trial, app, mobile phone, mHealth

## Abstract

**Background:**

Tobacco smoking remains the leading cause of preventable death and disease worldwide. Digital interventions delivered through smartphones offer a promising alternative to traditional methods, but little is known about their effectiveness.

**Objective:**

Our objective was to test the preliminary effectiveness of Quit Genius, a novel digital therapeutic intervention for smoking cessation.

**Methods:**

A 2-arm, single-blinded, parallel-group randomized controlled trial design was used. Participants were recruited via referrals from primary care practices and social media advertisements in the United Kingdom. A total of 556 adult smokers (aged 18 years or older) smoking at least 5 cigarettes a day for the past year were recruited. Of these, 530 were included for the final analysis. Participants were randomized to one of 2 interventions. Treatment consisted of a digital therapeutic intervention for smoking cessation consisting of a smartphone app delivering cognitive behavioral therapy content, one-to-one coaching, craving tools, and tracking capabilities. The control intervention was very brief advice along the Ask, Advise, Act model. All participants were offered nicotine replacement therapy for 3 months. Participants in a random half of each arm were pseudorandomly assigned a carbon monoxide device for biochemical verification. Outcomes were self-reported via phone or online. The primary outcome was self-reported 7-day point prevalence abstinence at 4 weeks post quit date.

**Results:**

A total of 556 participants were randomized (treatment: n=277; control: n=279). The intention-to-treat analysis included 530 participants (n=265 in each arm; 11 excluded for randomization before trial registration and 15 for protocol violations at baseline visit). By the quit date (an average of 16 days after randomization), 89.1% (236/265) of those in the treatment arm were still actively engaged. At the time of the primary outcome, 74.0% (196/265) of participants were still engaging with the app. At 4 weeks post quit date, 44.5% (118/265) of participants in the treatment arm had not smoked in the preceding 7 days compared with 28.7% (76/265) in the control group (risk ratio 1.55, 95% CI 1.23-1.96; *P*<.001; intention-to-treat, n=530). Self-reported 7-day abstinence agreed with carbon monoxide measurement (carbon monoxide <10 ppm) in 96% of cases (80/83) where carbon monoxide readings were available. No harmful effects of the intervention were observed.

**Conclusions:**

The Quit Genius digital therapeutic intervention is a superior treatment in achieving smoking cessation 4 weeks post quit date compared with very brief advice.

**Trial Registration:**

International Standard Randomized Controlled Trial Number (ISRCTN) 65853476; https://www.isrctn.com/ISRCTN65853476

## Introduction

### Background

Smoking is a leading cause of preventable and premature death worldwide. It is an important risk factor for serious health problems and life-threatening diseases [1]. Globally, tobacco use causes more than 8 million yearly deaths [2]. Moreover, the total global economic cost of smoking is more than $1.4 trillion a year [3]. Smoking is, therefore, a major worldwide economic and public health concern [1-3].

In the United Kingdom, very brief advice (VBA) is the recommended clinical practice for smoking cessation for all health care practitioners [4,5]. It is designed to promote quit attempts and to be used opportunistically in virtually any situation [4,5]. Those interested in quitting are referred to their local stop smoking service [4], which typically combines face-to-face behavioral support with the option of pharmacotherapy, offered as nicotine replacement therapy (NRT) or varenicline. Similarly, in the United States, smokers can access free telephone services and tobacco cessation websites that provide access to pharmacotherapy and additional behavioral support [6].

Despite traditional smoking cessation programs demonstrating efficacy, they only help about 8% of smokers to quit long-term [7]. Such programs have been shown to have limited utilization due to scheduling, time, and financial constraints [8,9]. Telephone support can overcome these barriers but reaches only about 1% of smokers annually [10]. Given that support may be difficult to access, there is an urgent need for alternative solutions that are cost-effective, convenient, and scalable.

Technological advancements have led to new approaches that aim to overcome the drawbacks of conventional smoking cessation programs. Smartphone apps are one new approach with the potential to support behavior change [11,12]. They have been used successfully across a multitude of therapeutic areas, including chronic conditions [13-15] and the promotion of healthy behaviors [16-22].

Smartphone apps have advantages over traditional approaches, including ease of accessibility, personalization of interactions with real-time feedback, scalability to large populations, and cost-effectiveness [23]. In 2018, the number of mobile phone subscriptions topped 8 billion globally [23]. Smartphone apps have the potential to reach smokers who would not or are unable to use traditional services.

However, there is a paucity of data that examine the efficacy of smartphone apps for smoking cessation. A review of mobile phone–based smoking support identified only 5 randomized controlled trials (RCTs) that tested the effectiveness of smartphone apps with low-intensity support, with each showing limited efficacy [23]. Furthermore, a recent content analysis revealed low adherence of existing smartphone apps to evidence-based treatment guidelines [24], while a review of the 50 most downloaded cessation apps found only 2 with scientific support [25].

Digital therapeutic interventions are a new wave of smartphone apps that can deliver high-intensity evidence-based therapeutic programs. Emerging evidence is encouraging and suggests that high-intensity support delivered via digital therapeutic interventions can aid smoking cessation. However, many promising early studies suffer from limitations, such as using single-arm cohorts or solely relying on self-reported abstinence without biochemical verification to assess intervention efficacy [19-22]. To date, very few RCTs have been conducted on digital therapeutic interventions for smoking cessation [26-28].

### Objectives

This study had several objectives. The first objective of this study was to test the preliminary effectiveness of the digital therapeutic intervention Quit Genius (QG) by measuring 7-day point prevalence abstinence at 4 weeks post quit date. Other objectives included assessing user engagement with QG, as well as testing its effect on cognitive, attitudinal, and emotional outcomes.

## Methods

### Design

We conducted a 2-arm, single-blinded, parallel-group, preregistered randomized controlled trial with 4-week, 6-month, and 12-month follow-up. This trial report is in accordance with the Consolidated Standards of Reporting Trials (CONSORT) checklist. Here we report the primary outcomes at 4 weeks only, as data collection for later time points is still ongoing. Approval was granted by the Health and Social Care Research Ethics Committee A (reference 18/NI/0171) and this research complies with the Declaration of Helsinki.

### Participants

Participants were recruited in the United Kingdom between January and November 2019. Adult smokers (aged 18 years or older) were invited to participate if they had been smoking at least 5 cigarettes a day for the past year, were not using any other form of stop smoking support, and had sufficient mobile phone functionality (fifth generation or higher for Apple iPhone or version 18 or higher for Android). The exclusion criteria were not speaking English, pregnancy, chronic obstructive pulmonary disease, psychiatric medication, and a serious health condition that would substantially hinder completion of the intervention or control, as determined by the study team. Participants with serious health conditions or using psychiatric medication were ruled out as a safety consideration.

Participants were recruited offline from primary care practices across London via SMS text messaging campaigns. Posters and leaflets at local community venues and advertisements on social media were also used. Recruitment advertisements and study information given to participants described the opportunity of being allocated to one of 2 possible behavioral interventions, in addition to optional NRT. No mention of a digital intervention was made to the participants before randomization. Participants randomized into the study received £10 (US $12.82) to offset travel expenses. Participants completed a questionnaire online or via the telephone at 4 weeks after their quit date and were paid £20 (US $25.63) for completion.

### Registration

The trial was registered in the International Standard Randomized Controlled Trial Number (ISRCTN) database (ISRCTN65853476) on December 18, 2018. On July 24, 2019, we adjusted the primary outcome to equate it with the majority of trials on smoking cessation. Specifically, the time window for assessing whether the participant had successfully quit was reduced from 2 weeks to 1 week. The previously approved primary outcome related to the Russell Standard (ie, self-reported abstinence in the past 2 weeks at 4 weeks post quit date). This was changed to capture self-reported 7-day point prevalence abstinence at 4 weeks. This decision was informed in part by a recent Delphi study, which found only partial compliance with the Russell Standard, as reported by smoking cessation experts [29]. Although we acknowledged a lack of consensus relating to outcome criteria in smoking cessation research, it was our opinion that the use of 7-day point prevalence is preferable to 14-day point prevalence, as it allows for greater comparability with other studies. Amending the primary outcome to 7-day point prevalence allowed for greater comparison with other studies of face-to-face, digital, and other low-intensity intervention smoking cessation trials (full justification is available on the ISRCTN page). This decision was made without having analyzed trial outcomes. The 14-day point prevalence was added as a secondary outcome. Another secondary outcome (number of quit attempts up to week 4 post quit date) is reported here as “any additional quit attempt after the quit date” because a continuous test was not appropriate on the distribution, which was predominantly 1 quit attempt. All other adjustments to the trial registration only concerned start and end dates of recruitment and publication to account for unexpected challenges in recruitment.

### Randomization and Masking

Participants were randomized 1:1 (treatment:control) using a block size of 4 participants through the trial management system Curebase (Curebase Inc) [30]. Researchers randomizing participants were blind to allocation until they had performed the randomization.

### Procedures

At first contact (via phone or online), participants were provided with study information and completed a questionnaire to determine eligibility. If eligible, participants were invited to attend an in-person baseline session where eligibility was reconfirmed and informed consent and baseline data were collected. Participants were then randomized. All participants were recommended to set their quit date within 2 weeks of randomization, but this was not a mandatory requirement for study participation. All participants were offered NRT in addition to their allocated intervention. At 4 weeks post quit date, participants were invited to complete the follow-up survey (via phone or online).

### Treatment Intervention: Quit Genius

Quit Genius is a digital therapeutic intervention comprising a smartphone app informed by the principles of cognitive behavioral therapy (CBT) [31]. It is a year-long program designed to support the user both before and after their quit date. Quit Genius delivers intervention components that have demonstrated efficacy in promoting smoking cessation, including self-monitoring, goal setting, encouraging medication adherence, and providing feedback on progress. QG was developed over many iterations, including engagement with smokers, patient representatives, and scientific advisors. The app collects data on users through in-app metrics to help personalize the program. Metrics include usage, session completion, program completion, and quit date. Additional data are collected based on user participation and feedback following CBT exercises, providing information such as the user’s reasons for quitting smoking and the reasons why they continue to smoke.

Content is tailored to the user and delivered in the form of animated videos, audio sessions, reflective exercises, and quizzes. The user is prompted to complete a series of self-paced steps on their smoking cessation journey, with each new step (and content) unlocking only once the previous step has been completed. The program content is divided into 2 stages. The “Essentials” stage, in which the user is prompted to complete a series of different steps before their quit date, covers aspects such as preparing for the quit date, using nicotine replacement therapy, and thinking about the reasons for quitting. The “Sustain” stage, which the user completes once they quit smoking, focuses on the general principles of relapse prevention and helps the user to stay smoke free in the long-term. In the time leading up to their quit date, users are encouraged to monitor their smoking habits daily by logging the number of cigarettes smoked, their triggers (how they felt when they wanted to smoke), and the intensity of their craving. Once the user has quit smoking, they are encouraged to log whether they are currently smoking.

**Figure 1:**
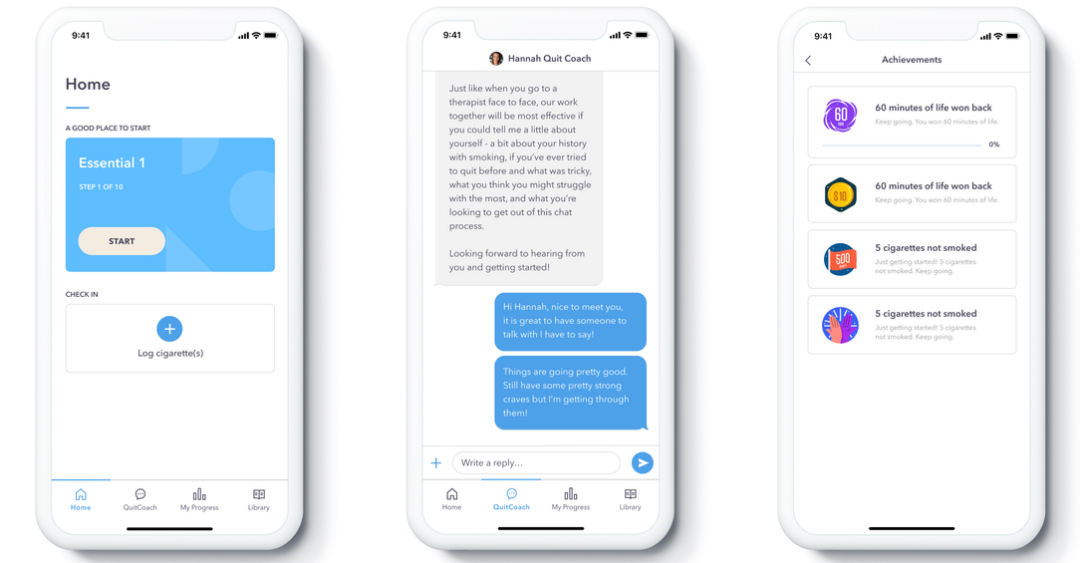
Screenshots of the digital therapeutic intervention Quit Genius.

As part of the QG digital therapeutic intervention, participants also have access to a quit coach, an advisor qualified by the National Centre for Smoking Cessation and Training (NCSCT). The coach provides personalized CBT-based support via a digital chat interface and the phone. Typically, participants partake in an initial phone call, with the rest of the quit coach interaction mediated through the in-app digital chat interface. Users can monitor their progress via the app, which details improvements to health and any financial benefits gained from being a nonsmoker since their quit date. Finally, users can access the “Craving Toolbox,” which comprises audio content of short breathing exercises, mindfulness exercises, and meditation exercises designed to help the user manage their cravings to smoke. The QG app uses CBT to target not only smoking cessation but also skills and strategies to promote improved mental health and well-being. While the QG app does not substitute professional care for mental health concerns, the app specifically addresses common mental health concerns such as low mood, anxiety, stress, self-esteem, and social skills. The app also specifically targets general health and well-being concerns, such as diet, exercise, and self-care techniques. Specific skills and techniques used include goal setting, cognitive restructuring, graded exposure, progressive muscle relaxation, mindfulness, assertiveness and communication training, and problem-solving skills. QG users receive push notifications to serve as reminders to engage with the app. All participants in the treatment group received free access to the QG intervention (screenshots shown in Figure 1).

### Control Intervention: Very Brief Advice

VBA is a simple form of advice designed to be used opportunistically. It follows the Ask, Advise, Act structure, as recommended by the UK government. Participants were advised to contact their local stop smoking service to access support and medication to quit smoking. Trial assistants were trained in the delivery of VBA, as per NCSCT guidelines. For the control group participants allocated a carbon monoxide (CO) device, a nonbranded mobile app (ASH app) was also provided to visualize CO readings for the participant. The control group mobile app was only used in conjunction with the CO device and contained no other content for participants.

### Nicotine Replacement Therapy

All participants had the option to receive nicotine replacement products (2-mg or 4-mg gum and 16-hour or 24-hour patches) free for 12 weeks, with the first 2-week supply issued at the baseline visit. Participants were allowed to purchase alternative forms of oral NRT.

### Carbon Monoxide Monitor

Half of all participants were given a CO monitor (Smokerlyzer; coVita Inc) to measure levels of carbon monoxide in their breath and to validate self-reported smoking abstinence. Participants were selected pseudorandomly to ensure 50% of each group was assigned a device. Devices were provided to 50% of participants for cost considerations and to explore if being assigned a CO monitor would affect quit rates between subgroups. CO levels were collected via self-reporting at 4 weeks post quit date. The CO devices plugged into the headphone or charging slot of participants' smartphones and were used in conjunction with the QG and control app. At the follow-up time point, participants were asked to give a reading from their device via the phone or online. NCSCT guidelines of a CO reading less than 10 ppm were used to validate participants' self-reported abstinence [32].

### Engagement

Engagement with the digital therapeutic intervention was measured via app opens, weeks actively using the app (defined as logging in to the app), stage progression through education and CBT components, number of messages sent between the participant and quit coach, check-ins (defined as a self-report of smoking status, that is, yes or no smoking after quit date), and diary entries (defined as registration of a cigarette before or after quit date using the in-app diary).

### Outcomes

Measurements were taken at baseline and at the 4-week follow-up. The following variables were collected at baseline: demographic details, smoking status, smoking history, expired carbon monoxide level, Fagerström Test for Nicotine Dependence [33], Smoking Abstinence Self-efficacy Questionnaire (SASEQ) [34], Warwick-Edinburgh Mental Well-being Scale (WEMWBS) [35], short version of the World Health Organization Quality of Life (WHOQOL-BREF) [36], and the service use questionnaire [37]. At 4 weeks post quit date, smoking status, changes in attitudes and perceptions of smoking, SASEQ, WEMWBS, and WHOQOL-BREF were collected. Expired carbon monoxide level was collected only in those participants who were assigned their own device. Measurements were collected via online questionnaires.

The primary outcome was self-reported 7-day point prevalence abstinence at 4 weeks post quit date. Secondary outcomes at week 4 were 14-day point prevalence abstinence, any additional quit attempts after the quit date, self-reported changes in confidence levels, knowledge, attitudes and perceptions related to smoking cessation, changes in SASEQ and WEMWBS, and satisfaction with the treatment intervention (treatment group only).

### Data Analysis

At 4 weeks, we expected to observe a 7-day abstinence rate of 25% in the treatment group and 10% in the control group. At a type I error rate of 5% and power of 90%, we required 133 participants per group (266 total). At 6 months, a conservative estimate would be a 10% quit rate in the treatment group and 3% quite rate in the control group. To detect a difference with 80% power and 5% type I error, we needed to randomize 194 participants per group (388 total). Assuming a 20% dropout, we aimed to recruit at least 500 participants.

We performed both intention-to-treat (ITT) [38] and per-protocol (PP) analyses. ITT included all participants assigned to treatment and control. ITT analysis assumed that participant data were not missing at random. PP included the subset of participants that provided answers to the self-reported outcomes at week 4.

We used chi-square tests for binary outcomes and 2-sample 2-tailed *t* tests for continuous outcomes. Those lost to follow-up at 4 weeks were considered as currently smoking for the primary outcome and 2-week abstinence. They were also considered as making no additional quit attempts after the quit date; not choosing “strongly agree” to improvements in confidence, knowledge, or attitude; and showing no change in self-efficacy or mental well-being from baseline. We used logistic regression to estimate the main effect of being assigned a CO device on likelihood of quitting, with treatment assignment as a covariate in the model. We made no corrections for multiple comparisons.

All data processing and analysis was performed in R (R Foundation for Statistical Computing) using the tidyverse package family and the fmsb package [39-41].

## Results

### Participant Flowchart

**Figure 2:**
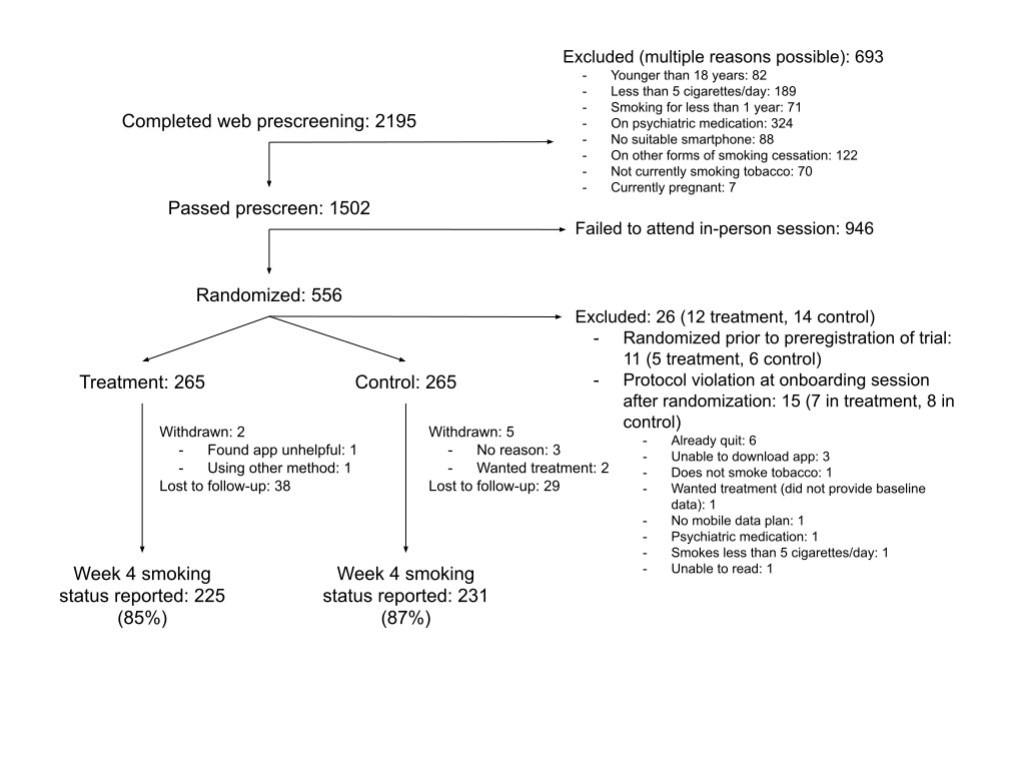
CONSORT flowchart.

Figure 2 shows the CONSORT flowchart for the RCT.

### Participants

A total of 556 participants were randomized (treatment: n=277; control: n=279). The intention-to-treat analysis included 530 participants (n=265 in each arm; 11 excluded for randomization before trial registration and 15 for protocol violations at baseline visit). Participants came from a wide age range (treatment: 19-73 years; control: 20-78 years), there were slightly more men than women, and about 2 in 3 self-identified as White (Table 1). Educational attainment ranged uniformly from secondary education to postgraduate education, and 80.0% (424/530) were in paid employment, of which over half were in managerial or professional roles. Participants were smoking on average 14 (treatment) or 15 (control) cigarettes per day, with nicotine dependence of 4 out of 10 on the Fagerström Test for Nicotine Dependence. Most participants (451/530, 85.0%) had previously made quit attempts, primarily by going “cold turkey” or with the help of e-cigarettes and NRT. No substantial differences between treatment and control groups were introduced through randomization.

**Table 1:** Demographics and smoking history of treatment and control groups.

Characteristic		Treatment	Control
Participants, n		265	265
Age (years), mean (SD)		40 (12)	42 (12)
Female, n (%)		123 (46.4)	116 (43.8)
**Ethnicity, n (%)**			
	White	183 (69.1)	164 (61.9)
	Black/Caribbean/African	25 (9.4)	30 (11.3)
	Asian	18 (6.8)	25 (9.4)
	Arab	3 (1.1)	4 (1.5)
	Mixed	24 (9.1)	23 (8.7)
	Other	7 (2.6)	11 (4.2)
	Prefer not to say	5 (1.9)	8 (3.0)
**Education, n (%)**			
	GCSE^a^ or lower	57 (21.5)	61 (23.0)
	A-level	65 (24.5)	51 (19.2)
	Undergraduate degree	76 (28.7)	77 (29.1)
	Postgraduate degree	44 (16.6)	51 (19.2)
	PhD	6 (2.3)	2 (0.8)
	Prefer not to say	17 (6.4)	23 (8.7)
In paid employment, n (%)		209 (78.9)	214 (80.8)
**Type of employment (if employed), n (%)**			
	Managerial or professional	126 (60.5)	113 (53.0)
	Routine or manual	22 (10.5)	33 (15.3)
	Intermediate	21 (10.0)	20 (9.3)
	Other	38 (18.1)	41 (19.1)
	Prefer not to say	2 (1.0)	7 (3.3)
Cigarettes per day, mean (SD)		14 (6)	15 (7)
Fagerström Test for Nicotine Dependence (range 0-10), mean (SD)		4 (2)	4 (2)
Any past attempt to quit smoking, n (%)		223 (84.2)	228 (86.0)
**Method previously used (if past attempts), n (%)**			
	Cold turkey	104 (46.8)	113 (49.4)
	E-cigarettes	93 (41.5)	95 (41.5)
	NRT^b^	68 (30.6)	64 (27.9)
	Prescription medication	24 (10.9)	34 (15.1)
	Smartphone app	20 (9.1)	22 (9.8)
	Hypnotherapy	8 (3.8)	16 (7.2)
	Psychological therapy	3 (1.5)	4 (1.9)

^a^GCSE: General Certificate of Secondary Education.

^b^NRT: nicotine replacement therapy.

### Engagement

Engagement with different facets of the digital therapeutic intervention is shown in Table 2. By the quit date (an average 16 days after randomization), 89.1% (236/265) of those in the treatment arm were still actively engaged. At the time of primary outcome, 74.0% (196/265) of participants were still engaging with the app. The content consisting of education and cognitive behavioral therapy (Essentials 1 and 2) were completed by 55.1% (146/265) and 35.8% (95/265) of participants, respectively. In addition, 69.1% (183/265) of participants sent at least one in-app message to their coach, and on average, people messaged their coach about once per week. On average, participants reported 12 diary entries to report cigarettes smoked before their quit date and 6 check-ins to report cravings or lapses after their quit date.

**Table 2:** Engagement with the digital therapeutic intervention in the treatment group (intention-to-treat participants, n=265).

Engagement	Value
App opens up to week 4, mean (SD), IQR	37 (52), 9-43
Days between randomization and quit date, mean (SD), IQR	16 (21), 9-23
Still active by quit date, n (%)	236 (89.1)
Still active 4 weeks after quit date, n (%)	196 (74.0)
Completed Essentials 1^a^, n (%)	146 (55.1)
Completed Essentials 2^b^, n (%)	95 (35.8)
Sent 1+ messages to coach, n (%)	183 (69.1)
Messages to coach before quit date, mean (SD), IQR	3.3 (5.9), 0-4
Messages to coach from quit date to week 4, mean (SD), IQR	4.2 (7.5), 0-6
Messages from coach before quit date, mean (SD), IQR	6.6 (6.3), 2-9
Messages from coach from quit date to week 4, mean (SD), IQR	6.2 (5.8), 2-9
Number of diary entries^c^ before quit date, mean (SD), IQR	12 (27), 1-12
Number of check-ins^d^ from quit date to week 4, mean (SD), IQR	6 (10), 0-9

**^a^**Essentials 1: program content aimed at preparation for the quit date.

^b^Essentials 2: program content intended for just after the quit date.

^c^Diary entry: registration of a cigarette smoked before and after the quit date.

^d^Check-in: self-report of smoking status after the quit date.

### Outcomes

Table 3 shows the primary outcome and secondary outcomes for intention-to-treat and per-protocol analyses. In the intention-to-treat analysis, those in the treatment arm were 55% more likely to report 7-day abstinence 4 weeks after their quit date compared to those in the control group (risk ratio 1.55, 95% CI 1.23-1.96; 118/265, 44.5% vs 75/265, 28.3% quit rate). In participants that were pseudorandomly assigned a CO device (treatment: 138/265; control: 142/265), 97.1% (134/138) in treatment and 97.9% (139/142) in the control group provided a CO reading at baseline, and 60.9% (84/138) in treatment and 66.9% (95/142) in the control group provided a reading at 4 weeks (including those who did not complete the week 4 questionnaire). CO completion was 88% (50/57) and 89% (33/37), respectively, in participants that claimed abstinence at 4 weeks. For these abstaining participants, the CO measurement was below 10 ppm for 96% (48/50) and 97% (32/33) of participants in treatment and control, respectively. Whether or not a participant was provided with a CO device did not significantly predict quit rate (*P*=.29 in logistic regression with CO device and intervention main effects). There was no difference in NRT use in treatment (133/225, 59.1%) and control (146/231, 63.2%) in those that completed the week 4 questionnaire (risk ratio 0.94, 95% CI 0.81-1.08), nor in electronic cigarette use in treatment (29/225, 12.9%) and control (23/231, 10.0%) groups (risk ratio 1.29, 95% CI 0.77-2.17).

**Table 3:** Outcomes at 4 weeks after quit date.

Outcome and group			Treatment	Control	Chi-square test (*df*)	*t* test (*df*)	*P* value	RR^a^ (95% CI)	Group difference (95% CI)
**Primary outcome**									
	**7-day abstinence, n (%)**								
		ITT^b^ (n=530)	118 (44.5)	75 (29.3)	13.7 (1)	N/A^c^	<.001	1.55 (1.23 to 1.96)	N/A
		PP^d,e^ (n=456)	118 (52.4)	75 (32.5)	17.8 (1)	N/A	<.001	1.62 (1.29 to 2.02)	N/A
**Secondary outcomes**									
	**14-day abstinence, n (%)**								
		ITT (n=530)	91 (34.3)	62 (23.3)	7.2 (1)	N/A	.007	1.47 (1.12 to 1.93)	N/A
		PP^e^ (n=456)	91 (40.4)	61 (26.4)	9.5 (1)	N/A	.002	1.53 (1.17 to 2.0)	N/A
	**Any additional quit attempt beyond initial quit date, n (%)**								
		ITT (n=530)	68 (25.6)	86 (32.5)	2.6 (1)	N/A	.10	0.79 (0.6 to 1.03)	N/A
		PP^f^ (n=443)	68 (30.9)	86 (38.6)	2.5 (1)	N/A	.11	0.8 (0.62 to 1.04)	N/A
	**Knowledge improved, n (%)^g^**								
		ITT (n=530)	104 (39.2)	77 (29.1)	5.7 (1)	N/A	.02	1.35 (1.06 to 1.72)	N/A
		PP^f^ (n=443)	104 (47.3)	76 (34.1)	7.5 (1)	N/A	.006	1.39 (1.1 to 1.75)	N/A
	**Confidence improved, n (%)^g^**								
		ITT (n=530)	84 (31.6)	65 (24.5)	3.0 (1)	N/A	.08	1.29 (0.98 to 1.7)	N/A
		PP^f^ (n=443)	84 (38.2)	64 (28.7)	4.1 (1)	N/A	.04	1.33 (1.02 to 1.74)	N/A
	**Attitude improved, n (%)^g^**								
		ITT (n=530)	100 (37.7)	103 (38.8)	0.0 (1)	N/A	.86	0.97 (0.78 to 1.21)	N/A
		PP^f^ (n=443)	100 (45.5)	101 (45.3)	0.0 (1)	N/A	>.99	1 (0.82 to 1.23)	N/A
	**Change in** **SASEQ^h^** **(24-point scale), mean (SD)**								
		ITT (n=530)	4.2 (7.0)	3.1 (6.7)	N/A	1.7 (527)	.09	N/A	1.0 (–0.161 to 2.17)
		PP^i^ (n=440)	5.1 (7.4)	3.6 (7.0)	N/A	2.0 (436)	.04	N/A	1.4 (0.052 to 2.75)
	**Change in WEMWBS^j^** **(56-point scale), mean (SD)**								
		ITT (n=530)	0.7 (7.0)	0.6 (6.5)	N/A	0.2 (525)	.83	N/A	0.13 (–1.02 to 1.28)
		PP^i^ (n=440)	0.9 (7.7)	0.8 (7.0)	N/A	0.2 (433)	.88	N/A	0.11 (–1.26 to 1.48)
**Unregistered outcomes**									
	**Cigarettes per day in those that failed to quit, mean (SD)**								
		PP (n=222)	7.9 (6.4)	7.5 (5.9)	N/A	0.5 (180)	.65	N/A	0.39 (–1.29 to 2.06)
	**% reduction in cigarettes per day in those that failed to quit, mean (SD)**								
		PP (n=222)	48.1 (28.2)	48.9 (29.3)	N/A	–0.2 (194)	.83	N/A	–0.83 (–8.57 to 6.91

^a^RR: risk ratio.

^b^ITT: intention to treat.

^c^N/A: not applicable.

^d^PP: per protocol.

^e^Treatment: n=225; control: n=231.

^f^Treatment: n=220; control: n=223.

^g^As measured by percentage of participants reporting “strongly agree.”

^h^SASEQ: Smoking Abstinence Self-efficacy Questionnaire.

^i^Treatment: n=219; control: n=221.

^j^WEMWBS: Warwick-Edinburgh Mental Well-being Scale.

For secondary outcomes, 14-day abstinence showed greater efficacy of treatment compared with control (risk ratio 1.47, 95% CI 1.12-1.93). Those in treatment were no more or less likely to have made an additional quit attempt after the initial quit date (risk ratio 0.79, 95% CI 0.60-1.03). Those in the treatment arm were more likely to strongly agree that their knowledge of their smoking habit had improved (risk ratio 1.35, 95% CI 1.06-1.72), but no such effect was observed regarding their confidence in their ability to stay smoke free (risk ratio 1.29, 95% CI 0.98-1.70) or in terms of whether their attitude toward stopping smoking had become more positive (risk ratio 0.97, 95% CI 0.78-1.21). The treatment was also not superior to control in terms of the increase in smoking self-efficacy (*P*=.09) or mental well-being (*P*=.83). However, in the per-protocol analysis, which included only participants that completed their week 4 outcomes, several secondary outcomes were significantly better in treatment compared with control; confidence improved more (risk ratio 1.33, 95% CI 1.02-1.74), as did self-efficacy (*P*=.04).

Participant satisfaction with the treatment intervention was high (213/265 in the treatment group that completed the questionnaire at week 4). On a scale from 0 (least satisfied) to 3 (most satisfied), mean quality of the smoking cessation service was 2.4 (SD 0.7), program meeting needs was 2.2 (SD 0.8), helpfulness of information was 2.6 (SD 0.6), helping to deal with smoking effectively was 2.4 (SD 0.7), and likelihood of coming back if needing to quit in the future was 2.5 (SD 0.7). When asked whether the participant would recommend the digital therapeutic intervention to a friend, 92.0% (196/213) would do so. Most participants considered the quit coach (72/213, 33.8%) and the education and CBT content (62/213, 29.1%) to be the most helpful. Some participants considered self-monitoring (28/213, 13.1%), the smoking diary (19/213, 8.9%), the craving toolbox (15/213, 7.0%), or other features (13/213, 6.1%) to be the most helpful. The community element of the digital therapeutic intervention was considered the least helpful, with only 1.9% (4/213) of participants considering it as the most helpful.

Finally, we examined 2 outcomes unregistered at trial registration. First, cigarettes smoked per day by those who failed to quit showed no difference between treatment and control groups (treatment: 7.9 cigarettes per day; control: 7.5 cigarettes per day). Similarly, the average percent decrease in cigarettes per day, though substantial in both groups, showed no difference between groups (48.1% decrease in treatment vs 48.9% decrease in control; *P*=.83).

## Discussion

### Principal Findings

In this RCT, we assessed the preliminary efficacy of Quit Genius, a digital therapeutic intervention for smoking cessation. The primary outcome of self-reported 7-day abstinence at 4 weeks post quit date was significantly higher (*P*<.001) for the treatment group compared with a control group using VBA.

Principally, this shows the superiority of the digital therapeutic intervention compared with the United Kingdom’s typical first-line intervention for smoking cessation [4,5]. We show that the digital therapeutic intervention is an effective method for short-term behavior change. The treatment group demonstrated a pseudorandomly CO-verified 4-week quit rate comparable with high-intensity face-to-face smoking cessation programs used by health care services in the United Kingdom [42]. Our results support previous literature illustrating that high-intensity behavioral support combined with pharmacotherapy is an effective means of quitting smoking [42].

Compared with other digital therapeutic interventions, the 44.5% (118/265) and 52.4% (118/225) 7-day abstinence rates 4 weeks after the quit date (for ITT and PP, respectively) compare favorably with previous digital intervention studies. Pivot, another digital therapeutic intervention, achieved an end-of-study CO-verified 7-day abstinence rate of 32% (ITT) and 37% (PP) [20]. An acceptance and commitment therapy intervention by Smartquit achieved 21% 7-day abstinence at 2 months post enrollment [22]. Clickotine’s 7-day abstinence rate of 45% at 8 weeks post enrollment was similar to that observed in the current study [21]. However, all these studies used single-arm designs rather than RCTs and were therefore unable to distinguish the causal effect of treatment, placebo, or underlying differences in propensity to quit in the study population [43]. Finally, both Smartquit and Clickotine studies used non–CO-verified self-reported abstinence as their primary means of assessing intervention efficacy, leaving a possibility of falsely reported smoking status [21,22]. This study largely avoided such limitations by using a 2-arm parallel-group RCT design with pseudorandom biochemical verification. While this study pseudorandomly verified the CO of ITT and PP participants, we found a near-perfect agreement (80/83, 96%) between the CO monitor and self-reported abstinence, suggesting that self-report can be taken at face value. The preliminary abstinence rates of the digital therapeutic intervention studied here are promising both in absolute terms and compared with the control group. Nevertheless, 6- and 12-month abstinence rates will be needed to confirm whether the intervention is also efficacious in the longer term.

### Cognitive, Attitudinal, and Emotional Improvements

Education and confidence are integral mechanisms in eliciting successful smoking cessation [44,45]. In this study, treatment caused a greater improvement in knowledge of personal smoking habits compared with control. Similarly, confidence in ability to stay smoke free was higher in treatment than control, though only statistically significant in participants that completed the study per protocol (*P*=.04). No effect was found on the attitude of participants toward stopping smoking. This suggests that the digital therapeutic intervention is an effective tool for making people aware of their habit and instilling some degree of confidence but fails to improve a commonly negative attitude toward stopping smoking. Given that smoking cessation interventions may be enhanced by incorporating strategies that target attitude change [46], the intervention could be further improved to engender a more positive attitude toward quitting.

Self-efficacy is a robust indicator of future successful smoking abstinence [45]. We observed the digital therapeutic intervention to be superior to the control in improving self-efficacy in participants who completed the study per protocol but not when analyzed by intention to treat. This resembles the increase in reported confidence, indicating that the digital therapeutic intervention enhanced users' beliefs in their capacity to quit successfully but that further developments on the intervention should focus on strengthening these outcomes.

We observed no benefit of the digital therapeutic intervention compared with the control in terms of mental well-being. In a retrospective study of the same intervention, we observed a correlation between hedonic well-being and quit rates [47]. In line with this, smoking cessation is typically associated with improved mental health, with evidence illustrating a reduction in anxiety, depression, and stress after quitting [48]. Given the higher quit rates in the treatment group, we expected treatment to be superior in terms of mental well-being. However, improvements are usually demonstrated in longer-term follow-ups than the 4 weeks reported here [48]. As such, changes in mental well-being might not yet have manifested, and our 6-month and 12-month outcomes will shed light on the longer-term impact of the digital therapeutic intervention on mental well-being. There are both positive and negative effects associated with smoking and smoking abstinence that could impact mental well-being. Evidence suggests that smokers who reduce their smoking but fail to quit show more pronounced mood deterioration than those who succeed [48]. Conversely, there is evidence illustrating the negative sequelae of smoking cessation, such as anxiety, insomnia, and weight gain [49]. Therefore, it is possible that simultaneous effects could have been active within groups, resulting in no net effect on mental well-being.

Nicotine addiction is a condition that rarely exceeds a lifetime abstinence rate greater than 50% per treatment. For this reason, many smokers take 30 or more quit attempts before being successful [50]. Nonetheless, a reduction in daily cigarette use predicts future behavior change; individuals who reduce their cigarette use by about 50% are more likely to see future quit attempts and greater odds of successful quitting [50]. Among treatment participants who had not quit smoking, there was an average reduction of 48% in their cigarette use compared to baseline, similar to control. Digital therapeutic interventions can, perhaps more easily than traditional programs, leverage such data to continue to engage and encourage specific participants after a failed quit attempt, identifying an optimal time to engage when chances of quitting are highest.

### Engagement

To elicit the success of any noninvasive digital therapeutic intervention, users must actively partake in the treatment [15,22]. We assessed engagement across several elements of the intervention and observed 89.1% (236/265) of the participants using the app until their quit date, with 74.0% (196/265) still using the app 4 weeks after their quit date. One differentiating aspect of this digital therapeutic intervention from typical smartphone apps is the presence of human coaching. We found a consistent bidirectional flow of communication between quit coach and participant from baseline to quit date and from quit date to 4-week follow-up. Of all the elements of treatment offered by the intervention, coaching was considered the most helpful. This reflects previous notions that smoking cessation programs that use health coaching as a means of support are effective in eliciting successful smoking abstinence [51]. Thus, the high level of engagement observed in this study may reflect the combination of in-app human coaching and other engagement features, such as push notifications, check-ins, and keeping a diary.

### Strengths and Limitations

Particular strengths of this study are the randomized controlled design, preregistration of the trial and its outcomes, pseudorandom biochemical verification despite the remote nature of the intervention, and high 4-week follow-up rate. The key limitation of this study is the short follow-up period of 4 weeks. It is known that relapse occurs over a longer time frame [52-54], and the current findings do not speak to the digital therapeutic intervention’s ability to prevent longer-term relapse.

Another limitation is that participants may have exaggerated their self-reported smoking abstinence. To combat this, participants were informed from study outset that regardless of smoking abstinence, they could remain on the trial and would be eligible for remuneration. Another preventative measure was the use of a measurement device to assess CO levels in the exhaled breath of the individual. Due to cost considerations, these devices were provided pseudorandomly to 50% of each study arm. The near-perfect agreement (80/83, 96%) between the CO monitor and self-reported abstinence suggests that, at least in the context of this digital therapeutic intervention, self-report can be taken at face value.

A further limitation was that the digital therapeutic intervention was not compared with another multifaceted intervention. VBA was chosen due to its use as the United Kingdom’s typical first-line intervention for smoking cessation, and for those participants not assigned a CO device, no app was provided. Therefore, it is plausible that participants may have guessed that they were assigned the control intervention due its limited functions.

There were also several limitations of the study sample. While the exclusion of participants with serious health conditions or using psychiatric medication was enforced as a safety consideration, it limits the generalizability to a smoking population that largely has other health and psychiatric conditions. Additionally, it is important to acknowledge that the study largely consisted of a sample of White, educated, and employed participants. Participants were required to attend an in-person baseline visit, so they had to live within a commutable distance to London. Therefore, the participant sample used in this study may be more reflective of an urban population, limiting the generalizability to more rural and remote-based populations.

Lastly, the lack of experimenter blinding to participant group allocation may have introduced bias into data interpretation. To avoid this, standardized questionnaires, participant interaction scripts, and standard operating procedures were used across treatment groups so that any effect on participant outcome data was minimized. In addition, the trial outcome measures and sample size were preregistered, and data were only analyzed after data collection had been completed.

### Conclusions

A digital therapeutic intervention for smoking cessation was superior to very brief advice in achieving smoking cessation after 4 weeks in a pseudorandomly biochemically verified RCT. The digital therapeutic intervention examined here is an effective option for short-term smoking cessation. Participants were actively engaged and satisfied with the intervention. Nevertheless, opportunities exist to improve mental well-being and attitudinal outcomes. A critical open question pertains to the long-term efficacy, which will be reported in a subsequent paper.

## Appendix

Multimedia Appendix 1 CONSORT-EHEALTH checklist (V 1.6.1).

## Abbreviations

CBT: cognitive behavioral therapy
CO: carbon monoxide
CONSORT: Consolidated Standards of Reporting Trials
ISRCTN: International Standard Randomized Controlled Trial Number
ITT: intention to treat
NCSCT: National Centre for Smoking Cessation and Training
NRT: nicotine replacement therapy
PP: per protocol
QG: quit genius
RCT: randomized controlled trial
SASEQ: Smoking Abstinence Self-efficacy Questionnaire
VBA: very brief advice
WEMWBS: Warwick-Edinburgh Mental Well-being Scale
WHOQOL-BREF: short version of the World Health Organization Quality of Life
